# Enhancement of cation exchange and glucose binding capacity, flavonoids release and antioxidant capacity of Tartary buckwheat powder with ultrafine grinding

**DOI:** 10.3389/fnut.2023.1276017

**Published:** 2023-10-19

**Authors:** Xinhui Wang, Xue Zhang, Dongjie Zhang

**Affiliations:** ^1^College of Food Science, Heilongjiang Bayi Agricultural University, Daqing, Heilongjiang Province, China; ^2^Key Laboratory of Agro-Products Processing and Quality Safety of Heilongjiang Province, Daqing, Heilongjiang Province, China; ^3^National Coarse Cereals Engineering Research Center, Daqing, Heilongjiang Province, China

**Keywords:** Tartary buckwheat, ultrafine grinding, functional properties, flavonoid, *in vitro* digestion

## Abstract

The objective of this paper was to study the effects of ultrafine grinding on the cation exchange capacity, glucose binding capacity and *in vitro* digestion characteristics of Tartary buckwheat powder. The results showed that the cation exchange ability and glucose binding strength of Tartary buckwheat powder, Tartary buckwheat bran powder and Tartary buckwheat core powder increased significantly with the increase of crushing frequency (20, 40 and 60 Hz), and the Tartary buckwheat bran powder was the highest. The results of *in vitro* digestion showed that ultrafine grinding improved the flavonoid release and antioxidant activity of Tartary buckwheat bran powder in the *in vitro* digestion process. The correlation analysis indicated that the amount of flavonoids released in digestive fluid was significantly related to antioxidant activity. This study may provide a theoretical basis for improving the physicochemical properties and functions of Tartary buckwheat by ultrafine grinding technology.

## 1. Introduction

Tartary buckwheat is a common whole grain used in both medicine and food (1). Tartary buckwheat has a variety of nutrients and good medicinal and health functions, and was also an excellent functional food development raw material (2). Because Tartary buckwheat was rich in phenolics, vitamins, dietary fiber and other active substances, it plays a greater value in the prevention and treatment of a variety of diseases (3). Therefore, the processing and utilization of Tartary buckwheat has become a research hotspot in recent years.

Ultrafine grinding technology was used to produce micron, submicron and even nanometer (100 μm ~ 1 nm) size powder (4). Compared with traditional mechanical grinding methods, ultrafine grinding provided better physicochemical and functional properties, such as good powder fluidity and hydration properties, higher bioavailability and bioactivity, significant antioxidant activity, lower interfacial tension, and better flavor release and taste (5). Therefore, owing to its distinctive grinding technique and favorable powder properties, the ultrafine grinding technology exhibits promising prospects in the research and development of novel functional food products.

The effects of ultrafine grinding on the functional properties of foods powder were mainly reflected in the glucose binding capacity, cation exchange capacity, sodium cholic acid adsorption capacity and antioxidant activity. Previous study showed that the adsorption capacity of carrot insoluble dietary fiber to nitrite and lead ions was significantly improved after ultrafine grinding (6). Another study reported that the DPPH free radical scavenging ability and ABTS cationic free radical scavenging ability of *Lycium barbarum* polysaccharide were significantly improved after micro-pulverization (7). The orange insoluble dietary fiber with air flow ultrafine grinding treatment could significantly improve the cecum and fecal indexes of mice, reduce the ammonia concentration in cecum, increase the fecal water content, and reduce the fecal β-D-glucuronidase activity (8). Additionally, the antibacterial activity and bactericidal activity of star fruit fiber powder ultrafine grinding with were increased by 2–8 times and 2–4 times respectively, which promoted the application of ultrafine grinding in the field of food preservation and antibacterial (9). These studies showed that ultrafine grinding has a great promotion effect on improving the functional properties of food. However, the effect of ultrafine grinding on the *in vitro* digestion of Tartary buckwheat was rarely reported.

Currently, the majority of studies on Tartary buckwheat powder primarily focus on flavonoid extraction and basic food processing techniques, with a relatively limited range of research topics. Limited research has been conducted on the comparative analysis of Tartary buckwheat whole powder, Tartary buckwheat bran powder, and Tartary buckwheat core powder. Therefore, we will conduct ultrafine pulverization of Tartary buckwheat whole powder, bran powder, and core powder to determine their cation exchange capacity, glucose binding capacity, release of flavonoids, and antioxidant activity post-digestion. This study aims to provide theoretical references and data support for the advanced processing of Tartary Buckwheat powder.

## 2. Materials and methods

### 2.1. Materials

The Tartary buckwheat bran powder, Tartary buckwheat core powder, and Tartary buckwheat whole powder were purchased from Liangshan Jianmao Food Co., LTD, Sichuan, China. The fluidized bed jet mill equipment (LHL type) was provided by Shandong Weifang Zhengyuan Powder Engineering Equipment Co., LTD, Shangdong, China.

### 2.2. Preparation of buckwheat powder

The Tartary buckwheat bran powder, core powder and whole powder were placed in the drying oven at 50°C to dry until the moisture content was below 6%. After undergoing 100-mesh screening, the processed powders of Tartary buckwheat bran (TBP), Tartary buckwheat core (TCP), and Tartary Buckwheat whole (TWP) were obtained and stored at a temperature of 4°C. The feed volume of the fluidized bed jet mill equipment was 1.5 kg, the air pressure was 0.8 MPa, and the crushing time was 90 min. The three kinds of buckwheat flour were milled once at the frequency of 20, 40 and 60 Hz respectively, with 1 kg in each group. Nine kinds of buckwheat flour were obtained, named TBP-20, TBP-40, TBP-60, TCP-20, TCP-40, TCP-60, TWP-20, TWP-40, and TWP-60, respectively. All Tartary buckwheat powders were stored at 4°C until analyzed.

### 2.3. Particle size and specific surface area

The particle size and specific surface area of the prepared Tartary buckwheat powder were determined using a laser particle size analyzer (Bettersize 2000), repeat 3 times.

### 2.4. Determination of cation exchange capacity

The cation exchange capacity of samples were determinated according to the method of Zhao et al. (10) with minor modification. The sample (0.50 g) was placed in a conical bottle, and 100 mL 0.85 mol/L NaCl solution was added, and the pH value of the solution was determined after magnetic stirring at 37°C for 5 min. Then 0.1 mL 0.01 mol /L NaOH was added, and the pH value of the solution was determined after magnetic stirring for 5 min. Until the total volume of added NaOH reached 1.0 mL, the pH value of the solution was recorded successively, and the change trend of the added amount of NaOH and the pH value of the solution was shown by a line chart.

### 2.5. Determination of glucose binding capacity

The glucose binding capacity of samples were determinated following a method of Sangnark and Noomhorm (11) with slight modification. The 0.50 g sample was added into a conical bottle with 100 mL glucose solution of different concentrations (5, 10, 50, 100 mmol/L), stirred magnetically at 37°C for 6 h to fully absorb the glucose solution, centrifuged at 3773 g for 20 min, and the supernatant was taken to determine the glucose content (Glucose detection kit, O-toluidine method). The glucose binding capacity was calculated by formula (1).


(1) $$GAC=\frac{C_{0}-C_{1}}{m}\times 0.1$$

Where GAC is powder glucose binding capacity, mmol/g; C_0_ is the concentration of glucose solution before adsorption, mmol/L; C_1_ is the concentration of glucose in the supernatant after adsorption, mmol/L; m is powder mass, g.

### 2.6. *In vitro* simulated gastric digestion

The *in vitro* simulated gastric digestion were analyzed following a method reported by Li et al. (12) with slight modification. Dissolve the sample (5.0 g) in a conical bottle containing 100 mL of normal saline solution, followed by gradual addition of 0.5 mol/L HCL dropwise until achieving a pH value of 2, and then 10 mL simulated gastric juice (0.2 g pepsin was dissolved in 10 mL 0.01 mol/L HCl) was added. In the control group of gastric acid, the simulated gastric juice was replaced by 0.01 mol/L HCl solution of equal volume. In the blank control group, hydrochloric acid and simulated gastric juice were replaced with equal volume of normal saline, and the pH value of the blank control group was 7.

All conical bottle were wrapped in aluminum foil and placed in a constant temperature (37°C) and shock incubator, which was digested at 100 r/min for 2 h under anaerobic conditions, and sampled at 0, 0.5, 1, 1.5 and 2.0 h after gastric digestion. The samples were centrifuged at 4°C and 16,099 g for 10 min, and the supernatant was taken to determine the flavonoid content.

### 2.7. *In vitro* simulated intestinal digestion

The *in vitro* simulated intestinal digestion were evaluated using a method reported by Li et al. (13) with slight modification. After gastric digestion, 1 mol/L NaHCO_3_ was added to the solution drop by drop to make the pH value of the solution 7.0, and then 18 mL simulated intestinal fluid (0.1 g trypsin and 0.65 g bile extract were dissolved in 50 mL 0.1 mol/L NaHCO_3_) was added. The simulated intestinal fluid was replaced by equal volume of 0.1 mol/L NaHCO_3_ in the blank control group.

The conical bottle was wrapped in aluminum foil and placed in a constant temperature (37°C) shock incubator, which was digested at 100 r/min under anaerobic conditions for 2 h, and sampled at 0, 0.5, 1, 1.5 and 2 h for intestinal digestion. The samples were centrifuged at 4°C and 16,099 g for 10 min, and the supernatant was taken to determine the flavonoid content.

### 2.8. Determination of flavonoid content

The flavonoid content of samples were determinated according to the method of Li et al. (14) with minor modification. The supernatant (2 mL) was added to the test tube, and then 0.3 mL 5% NaNO_2_ solution was added, and then 0.3 mL 10% Al(NO_2_)_3_ solution was added, and then 4 mL 4% NaOH solution was added, and 75% ethanol solution was added to the scale and then shaken for 10 min. The absorbance value of sample was measured at 510 nm wavelength. The standard curve of rutin was drawn with the concentration of rutin standard substance as the horizontal coordinate (0, 50, 100, 150, 200 μg/mL) and the absorbance value as the vertical coordinate.

### 2.9. Antioxidant activity

#### 2.9.1. Hydroxyl radical scavenging ability

The hydroxyl radical scavenging ability of samples were determinated according to a method described by Giao et al. (15), with minor modification. The digestible solution (2 mL) was placed into a test tube, followed by 2 mL of 6 mmol/L FeSO_4_ and 2 mL of 6 mmol/L H_2_O_2_, mixed for 10 min, then 2 mL of 6 mmol/L salicylic acid-ethanol solution was added, mixed for 30 min. The absorption value was measured at 510 nm. Distilled water was used to replace samples for blank control, and distilled water was used to replace H_2_O_2_ for sample control. The scavenging ability of hydroxyl free radicals was calculated by formula (2).


(2) $$K=\left(1-\frac{A_{1}-A_{2}}{A_{0}}\right)\times 100\%$$

Where K is the scavenging ability of digestive fluid on hydroxyl free radicals, %; A_1_ is the absorption value of the sample to be tested; A_2_ is the absorption value of the sample to be measured when distilled water replaces H_2_O_2_ in the reaction; A_0_ is the absorption value of distilled water when the sample participates in the reaction.

#### 2.9.2. DPPH free radical scavenging ability

The DPPH free radical scavenging ability of samples were analyzed according to a method reported by Li et al. (16), with minor modification. The 2 mL of digestive fluid was put into a test tube, and 4 mL of 0.1 mmol/L DPPH-ethanol solution was added. After mixing, it was placed at 25°C for 30 min away from light, and its absorption value was measured at 510 nm. The sample was replaced by anhydrous ethanol for blank control, and the sample was replaced by anhydrous ethanol for DPPH-ethanol solution for sample control. The scavenging ability of DPPH free radicals was calculated by eq. (3).


(3) $$Y=\left(1-\frac{A_{i}-A_{j}}{A_{0}}\right)\times 100\%$$

Where Y is scavenging ability of digestive fluid on DPPH free radicals, %; A_i_ is absorption value of the sample to be tested; A_j_ is absorption value of the sample to be measured when anhydrous ethanol replaces DPPH-ethanol solution; A_0_ is the absorption value when anhydrous ethanol replaces the sample in the reaction.

#### 2.9.3. ABTS^+^ free radical scavenging ability

The ABTS^+^ cationic free radical scavenging ability of samples were evaluated according to a method reported by Ma et al. (17), with minor modification. The 7 mmol/L ABTS^+^ solution was mixed with 2.45 mmol/L potassium persulfate solution at 1:1 volume ratio and placed away from light for 16 h to obtain ABTS^+^ reserve solution. Before determination, ABTS^+^ reserve solution was diluted with methanol solution so that its absorption value at 734 nm was 0.70 ± 0.02 to obtain ABTS^+^ working solution. The mixed solution of 0.2 mL digestion solution and 3.8 mL ABTS^+^ was mixed and placed at 25°C for 30 min away from light. The absorption value was determined at 734 nm. The sample was replaced by methanol solution for blank control, and the sample was replaced by methanol for ABTS^+^ mixture for control. The scavenging ability of ABTS^+^ free radicals was calculated by eq. (4).


(4) $$R=\left(1-\frac{A_{m}-A_{n}}{A_{k}}\right)\times 100\%$$

Where R is the scavenging ability of digestive fluid to ABTS^+^ free radicals, %; A_m_ is absorption value of the sample to be tested; A_n_ is absorption value of the sample to be measured when methanol replaces ABTS^+^ mixed solution reaction; A_k_ is the absorption value of methanol when the sample participates in the reaction.

### 2.10. Statistic analysis

All test results were the mean of 3 trials, expressed as the mean ± standard deviation. The SPSS 26.0 software was used for data analysis, and Duncan method was used to test the significance of the difference, and *p* < 0.05 indicated significant difference, and Pearson algorithm was used for the correlation analysis.

## 3. Results and discussions

### 3.1. Particle size and specific surface area of Tartary buckwheat powder

**Figure 1:**
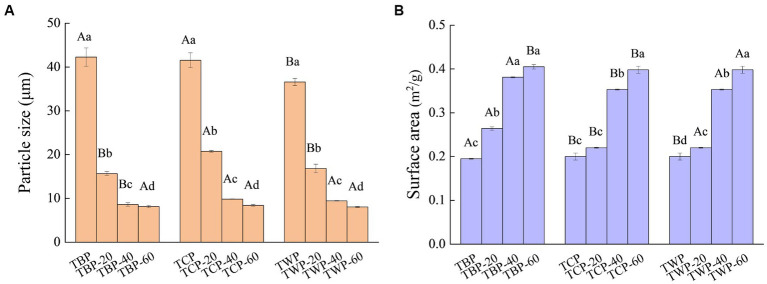
Particle size and specific surface area of Tartary buckwheat powder. **(A)**: Particle size of Tartary buckwheat powder, **(B)**: Surface area of Tartary buckwheat powder. Capital letters indicate: under the same ultrafine crushing conditions, different categories of Tartary buckwheat powder comparison, different letters differ significantly (*p* < 0.05). Small case letters indicate: under the same category of conditions, different ultrascopic crushing conditions of Tartary buckwheat powder comparison, different letters differ significantly (*p* < 0.05).

The particle size is a crucial physical parameter for the evaluation of grain powder samples, exerting a significant impact on both grain quality and processing efficiency (18). The particle size of TBP is smaller compared to TCP and TWP, as depicted in Figure 1A. After undergoing ultrafine grinding, the particle size of various types of Tartary buckwheat powder exhibited a significant reduction. Furthermore, as the frequency of ultrafine grinding treatment increased, the particle size reduction in Tartary buckwheat powder became even more pronounced (*p* < 0.05).The findings demonstrate the effective reduction of particle size in Tartary buckwheat powder through air flow ultrafine grinding, thereby exhibiting a favorable comminution effect on the Tartary buckwheat powder.

The variation in specific surface area significantly impacts the adsorption capacity of the micropowder towards solvents, rendering powder samples with high specific surface areas highly promising as potential food additives or active ingredients. The increase in the specific surface area of grain meal is advantageous for enhancing material contact, reducing reaction time, improving raw material utilization rate, increasing product yield, and optimizing product performance (19). The specific surface area of various types of Tartary buckwheat powder increased a gradual increase following air flow ultrafine grinding, as depicted in Figure 1B, with statistically significant differences observed (*p* < 0.05). Compared to Tartary buckwheat powder of the same type, the specific surface area of Tartary Buckwheat powder increases with an increase in air flow grinding frequency, and the optimal effect is achieved at a frequency of 60 Hz during air flow ultrafine grinding.

### 3.2. Effect of ultrafine grinding on cation exchange capacity of Tartary buckwheat powder

**Figure 2:**
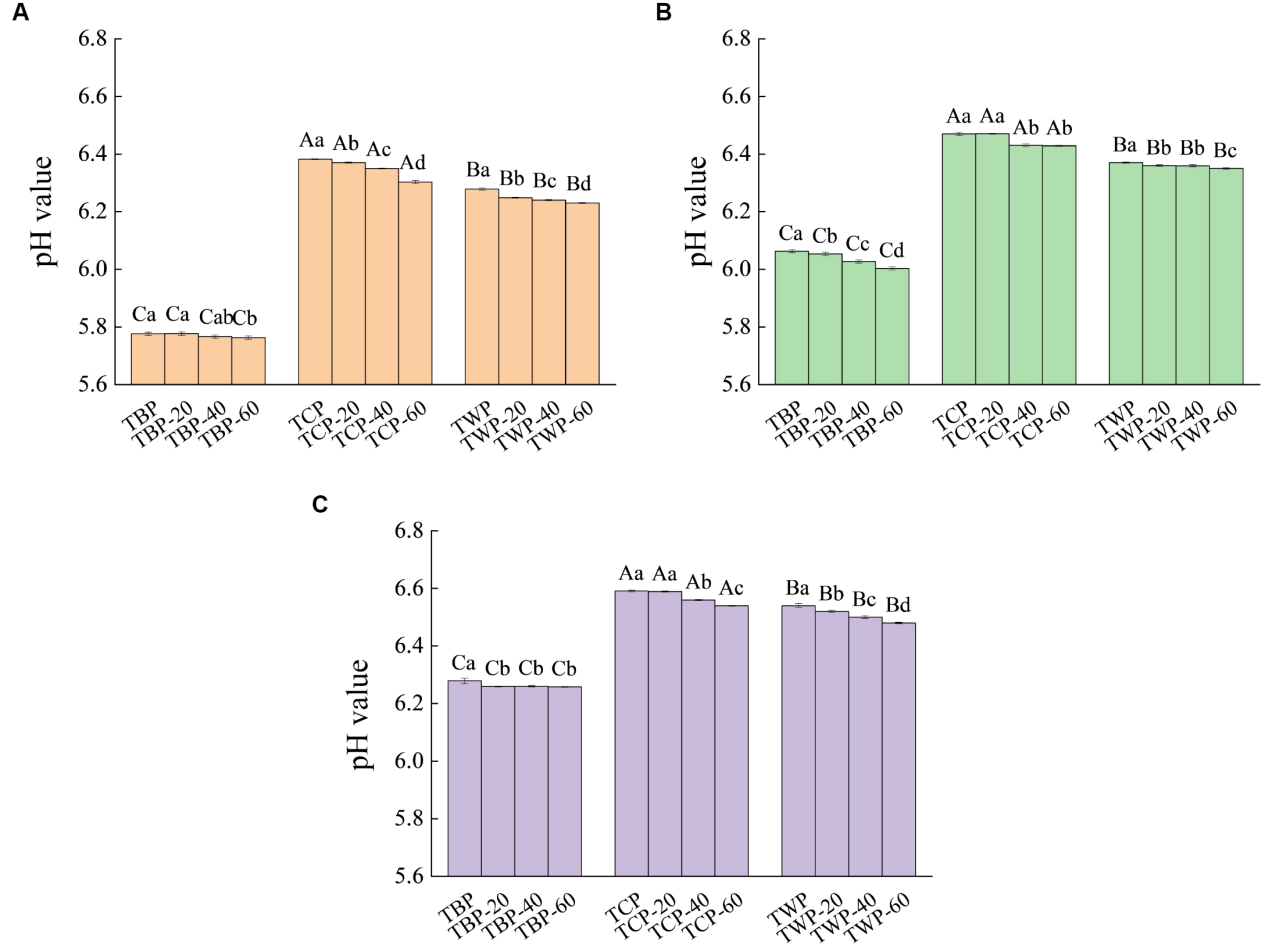
The effect of ultrafine grinding on cation exchange capacity of Tartary buckwheat powder. **(A)**: NaOH was added in an amount of 0 mL, **(B)**: NaOH was added in an amount of 0.5 mL, **(C)**: NaOH was added in an amount of 1.0 mL. Capital letters indicate: under the same ultrafine crushing conditions, different categories of Tartary buckwheat powder comparison, different letters differ significantly (*p* < 0.05). Small case letters indicate: under the same category of conditions, different ultrascopic crushing conditions of Tartary buckwheat powder comparison, different letters differ significantly (*p* < 0.05).

The effect of ultrafine grinding on cation exchange capacity of Tartary buckwheat powder is presented in Figure 2. As can be seen from Figure 2, different categories of Tartary buckwheat powder have certain cation exchange capacity. The smaller the pH value, the stronger its cation exchange capacity. The TBP and TWP have good cation exchange ability. The pH value of TBP-20, TBP-40, TBP-60, TWP-20, TWP-40, and TWP-60 solution increased gradually with the increase of the volume of NaOH. When the amount of NaOH was 1 mL, the pH value of each powder solution gradually became stable. Therefore, the cation exchange capacity of the powder could be improved by ultrafine grinding treatment, and the cation exchange capacity increased gradually with the decrease of particle size.

The powder with cation exchange ability can exchange Na^+^, K^+^ and other cations in the human gastrointestinal tract in a directional manner, reducing the ratio of Na^+^ and K^+^ in the blood, so that the gastrointestinal tract forms a relatively mild environment, conducive to human digestion and absorption (20). This was explained that the powder contained a certain amount of dietary fiber, they contained side chain groups such as carboxyl group and hydroxyl group which have the function of cation exchange resin, thus showing a certain adsorption effect (21). The TBP contained more dietary fiber structure, so the cation exchange capacity of TBP was higher than that of TWP and TCP. The cell wall was broken during the ultrafine grinding process, and the side chain groups such as carboxyl and hydroxyl groups in the powder particles were exposed, which enhanced the cation exchange ability (22). However, with the increase of pulverization degree, the cation exchange ability of the powder did not significantly improve. This could be explained that the increase of pulverization degree caused the long chain dietary fiber in the powder to break into broken chain dietary fiber, carboxyl group, hydroxyl group and other side chain groups were destroyed, and the adsorption ability was weakened (23).

### 3.3. Glucose binding capacity

**Figure 3:**
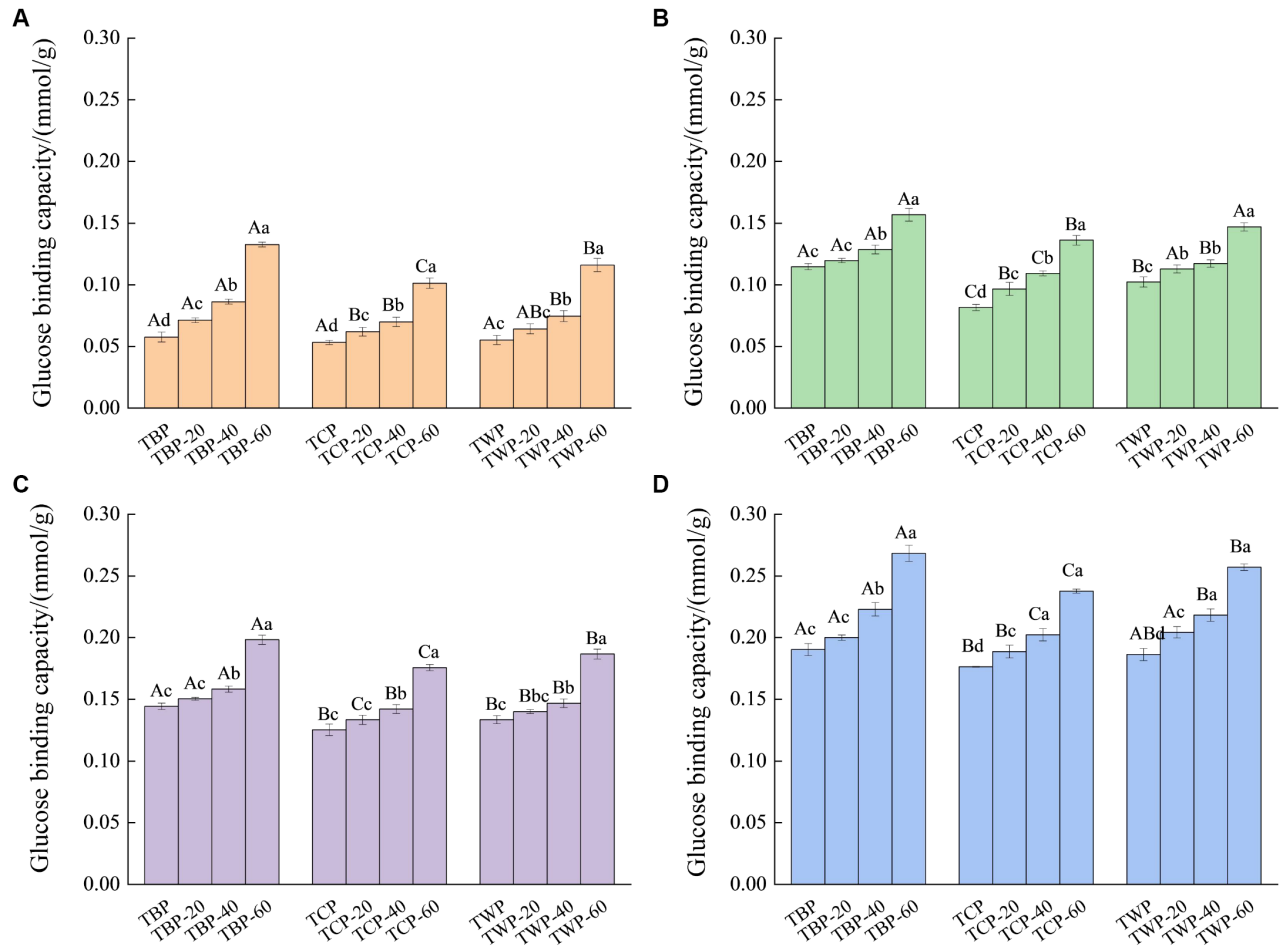
The effect of ultrafine grinding on glucose binding capacity of Tartary buckwheat powder. **(A)**: The initial glucose concentration is 5 mmol/L, **(B)**: the initial glucose concentration is 10 mmol/L, **(C)**: the initial glucose concentration is 50 mmol/L, **(D)**: the initial glucose concentration is 100 mmol/L. Capital letters indicate: under the same ultrafine crushing conditions, different categories of Tartary buckwheat powder comparison, different letters differ significantly (*p* < 0.05). Small case letters indicate: under the same category of conditions, different ultrascopic crushing conditions of Tartary buckwheat powder comparison, different letters differ significantly (*p* < 0.05).

The effect of ultrafine grinding on binding capacity of Tartary buckwheat powder to glucose is indicated in Figure 3. With the increase of initial glucose concentration, the glucose binding ability of Tartary buckwheat powder gradually increased, and the glucose binding capacity of bran powder was significantly higher than that of whole powder and core powder at the same concentration. The binding capacity of TBP at the initial glucose concentration of 5, 10, 50 and 100 mmol/L was 0.058, 0.115, 0.144 and 0.190 mmol/g, respectively. When the initial glucose concentration was from 5 mmol/L to 100 mmol/L, the binding capacity of TCP increased from 0.053 mmol/g to 0.176 mmol/g. Similarly, when the initial glucose concentration was from 5 mmol/L to 100 mmol/L, the binding capacity of TWP increased from 0.055 mmol/g to 0.186 mmol/g. Under the same initial glucose concentration, the glucose binding ability of Tartary buckwheat powder gradually increased with the increase of the crushing frequency. When the initial concentration of glucose was 50 mmol/L, the glucose binding capacity of TWP, TWP-20, TWP-40, and TWP-60 were 0.133, 0.140, 0.147, and 0.187 mmol/g, respectively. When the initial concentration of glucose was 100 mmol/L, the glucose binding strength of TBP, TBP-20, TBP-40, and TBP-60 were 0.190, 0.200, 0.223, and 0.268 mmol/g, respectively, and showed a gradual increasing trend.

The binding ability of TBP was higher than that of TWP and TCP under the same crushing conditions, which could be due to the high fiber content in TBP powder. The dietary fiber has high adsorbability, which enhanced the glucose binding ability of the TBP (24). Previous study reported that the particle size and surface area of whole wheat flour powder after air flow grinding were reduced, which significantly reduced the glycemic index of the products made from it (25). Additionally, another study showed that wheat bran dietary fiber could inhibit the diffusion of glucose after ultrafine grinding treatment (26). The ultrafine grinding treatment could increase the specific surface area of the powder, and make the spatial structure of the ultrafine powder loose, enhance the capillary effect, and promote the affinity adsorption and physical binding ability of dietary fiber to glucose molecules (27). Additionally, starch, protein and other substances could be bonded with glucose through hydrogen bonding, van der Waals force and other ways, thus improving the glucose adsorption capacity of the powder, which led to the Tartary buckwheat powder after ultrafine grinding has good glucose binding capacity (28). Therefore, the flavonoid content of TBP was higher than that of TWP and TCP, and it also had good cation exchange ability and glucose binding ability. Therefore, the TBP with different particle sizes was selected for follow-up *in vitro* simulated digestion test.

### 3.4. Changes in the flavonoid content of Tartary buckwheat bran powder during *in vitro* gastric digestion

**Figure 4:**
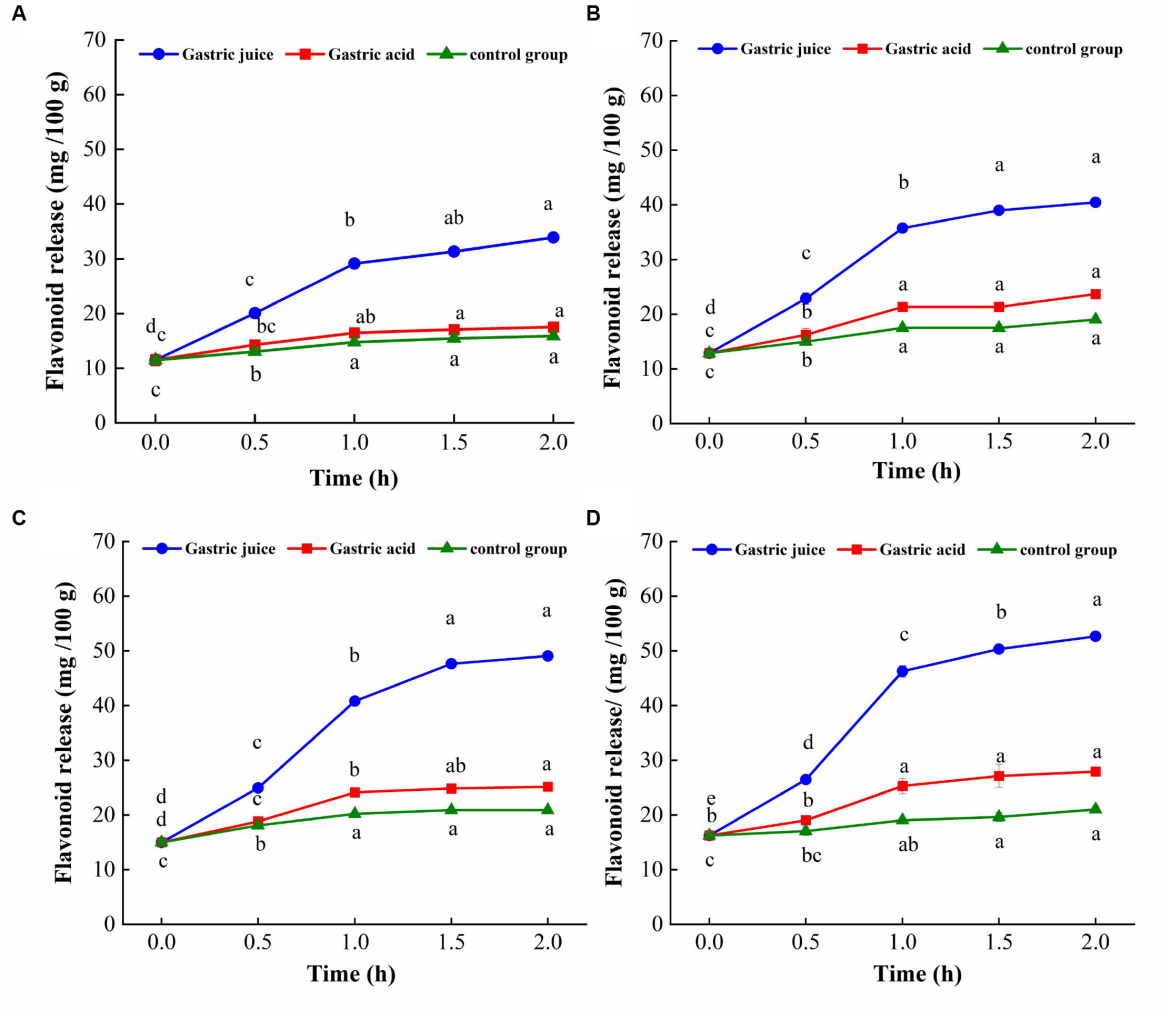
Flavonoid levels change of Tartary buckwheat bran powder with ultrafine grinding during *in vitro* gastric digestion. **(A)**: TBP; **(B)**: TBP-20; **(C)**: TBP-40; **(D)**: TBP-60. Small case letters indicate: under the same treatment conditions, different digestion time contrast, different letters differ significantly (*p* < 0.05).

The change trend of flavonoid content of TBP with different crushing frequency during *in vitro* gastric digestion is shown in Figure 4. The flavonoids release of TBP was significantly increased in the first 1 h of digestion, tends to stability within 1 to 2 h. The amount of flavonoids released by different crushing frequency of TBP was different. The flavonoid release of TBP was 11.48 mg/100 g at the initial stage of gastric digestion, and 33.88 mg/100 g at the end of gastric digestion. The flavonoid release of TBP-20 was 12.89 mg/100 g at the initial stage of gastric digestion, and 40.49 mg/100 g at the end of gastric digestion. The flavonoid release of TBP-40 was 14.98 mg/100 g at the initial stage of gastric digestion, and 49.07 mg/100 g at the end of gastric digestion. Similarly, the flavonoid release of TBP-60 was 16.26 mg/100 g at the initial stage of gastric digestion, and 52.55 mg/100 g at the end of gastric digestion. Compared with the initial stage of gastric digestion, the flavonoid release of TBP,TBP-20, TBP-40, and TBP-60 increased by 22.40, 27.60, and 36.29 mg/100 g at the end of gastric digestion, respectively.

The TBP treated with ultrafine grinding has good flavonoid release characteristics during *in vitro* stomach digestion, which may be because the specific surface area of the powder and the contact area between the powder and the solvent increase after ultrafine grinding, which may be conducive to the dissolution of the effective components (29). The reason why the release of flavonoids in the gastric digestion group gradually increased and was higher than that in the gastric acid control group and the blank control group may be because the pepsin in the simulated gastric fluid hydrolyzed the macromolecular proteins in TBP into small molecular peptides, thereby releasing some protein-coated flavonoids (30). Additionally, pepsin played a dominant role in the release of flavonoids during gastric digestion, so the amount of flavonoids released in digestive group was higher than that in control group and blank group.

### 3.5. Changes in the flavonoid content of Tartary buckwheat bran powder during *in vitro* intestinal digestion

**Figure 5:**
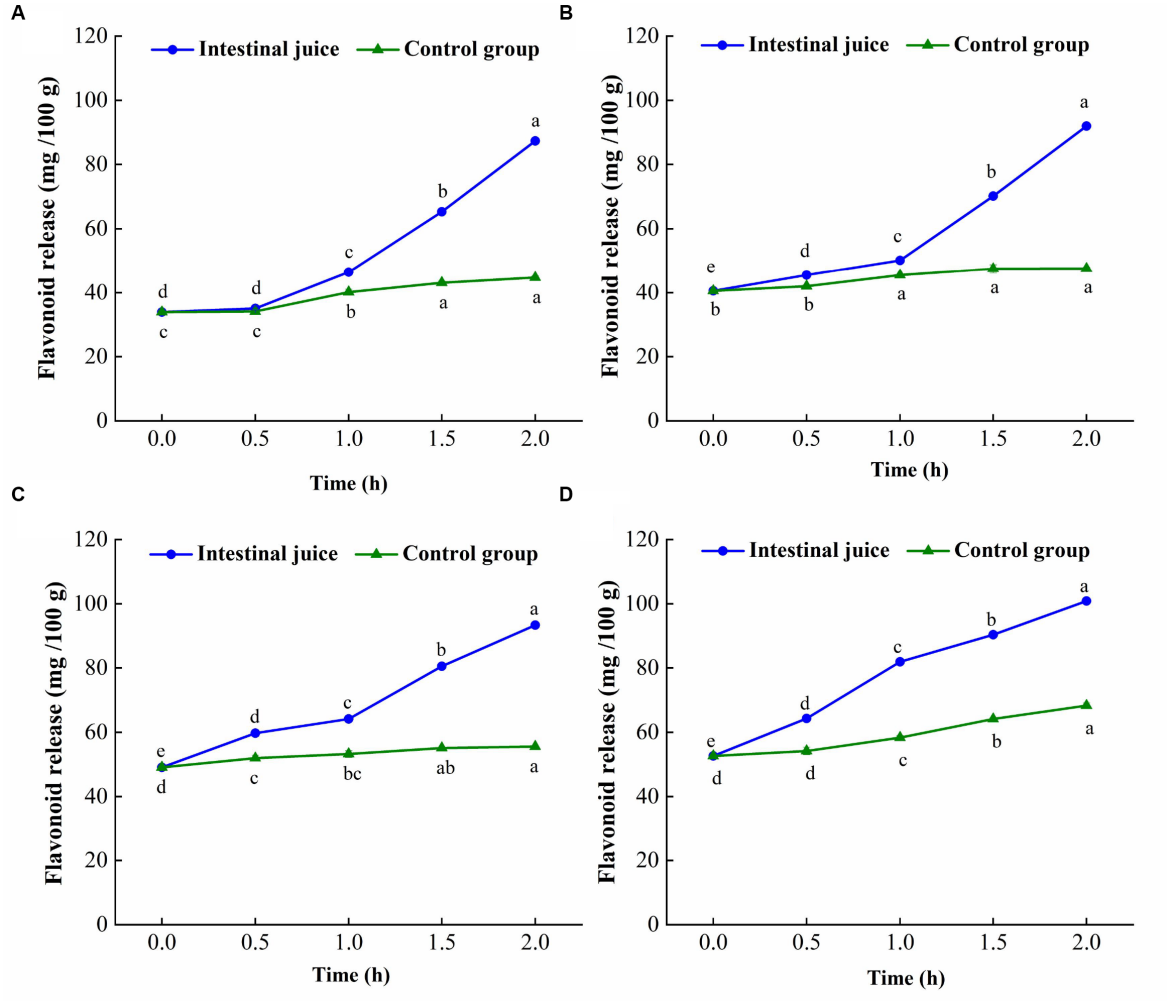
Flavonoid levels change of Tartary buckwheat bran powder with ultrafine grinding during *in vitro* intestinal digestion. **(A)**: TBP; **(B)**: TBP-20; **(C)**: TBP-40; **(D)**: TBP-60. Small case letters indicate: under the same treatment conditions, different digestion time contrast, different letters differ significantly (*p* < 0.05).

The change trend of flavonoid content of TBP with different crushing frequency during *in vitro* intestinal digestion is shown in Figure 5. The flavonoids release of TBP with different crushing frequency also showed an increasing trend during *in vitro* intestinal digestion. However, different from the gastric digestion stage, the flavonoid release of TBP was relatively stable in the first 0.5 h of intestinal digestion, and significantly increased within 1 to 2 h of digestion, and the release amount in *in vitro* intestinal digestion was higher than that in *in vitro* gastric digestion. The maximum flavonoids release of TBP in *in vitro* intestinal digestion stage was 87.33 mg/100 g, which was 2.58 times that of TBP without intestinal digestion. In the stage of intestinal digestion, the flavonoid release of TBP-20 increased from 40.49 mg/100 g to 91.97 mg/100 g, an increase of 2.27 times. In the stage of intestinal digestion, the flavonoid release of TBP-40 increased from 49.07 mg/100 g to 93.36 mg/100 g, an increase of 1.90 times. Similarly, the maximum flavonoid release of TBP-60 was 100.89 mg/100 g, which was 1.92 times higher than that in the initial stage of digestion. Additionally, the flavonoid release of TBP with different crushing frequency in intestinal fluid digestion group was significantly higher than that in blank control group.

Previous study have shown that with the increase of digestion time, the release of phenolics in brown rice with different milling degrees significantly increased, and the release of polyphenols in the intestinal stage was more than that in the gastric digestion (31). This was similar to the results of this study. The trypsin in the simulated intestinal fluid can break the carboxyl group of proteins that interact with flavonoids (32). Additionally, phenolic acids, anthocyanins and other substances degraded in a weakly alkaline environment, transforming their structure into a more stable flavonoid structure, thereby increasing the release of flavonoids (33).

### 3.6. Changes in the antioxidative activity of Tartary buckwheat bran powder during *in vitro* digestion

**Figure 6:**
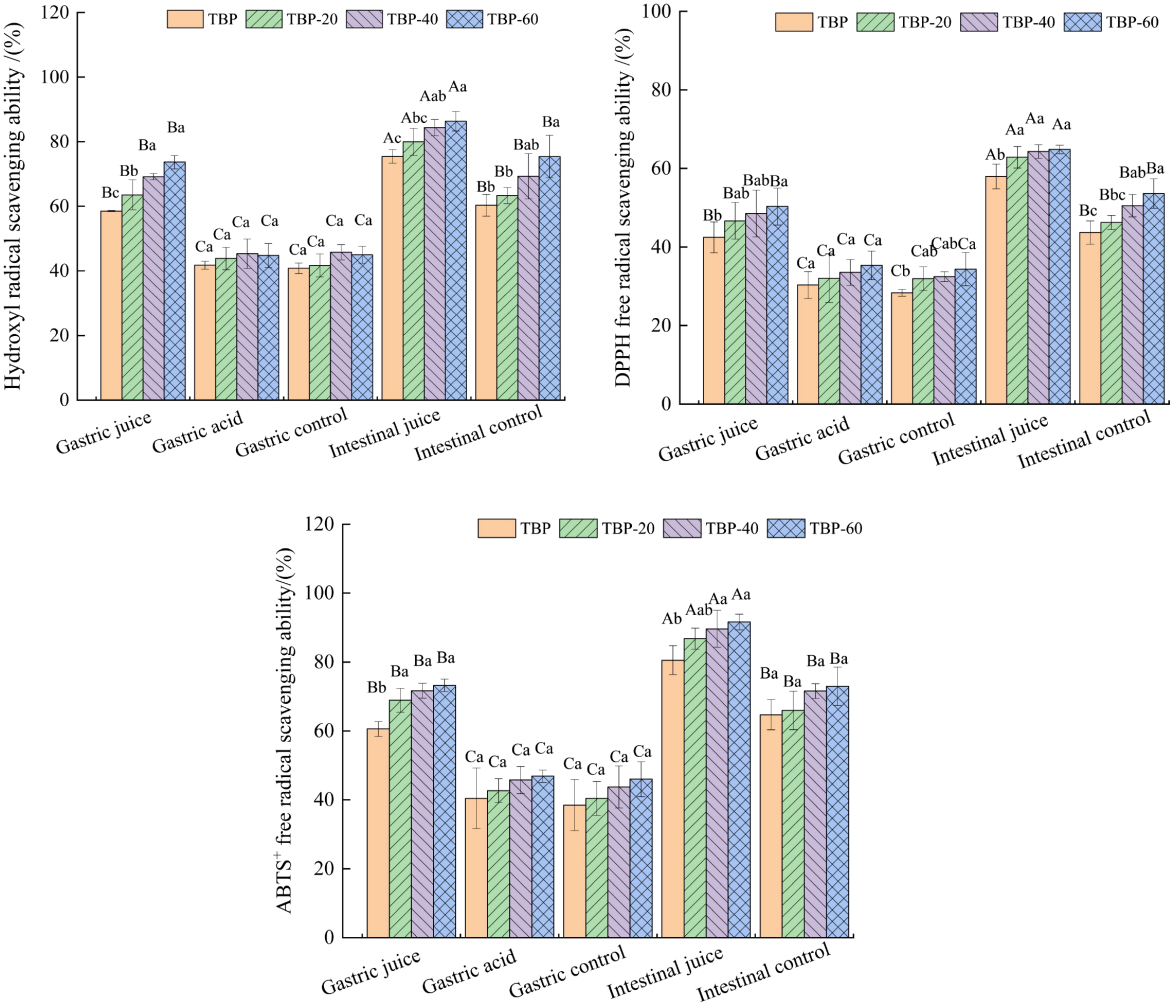
Changes in Hydroxyl radical, DPPH and ABTS^+^ scavenging activity of Tartary buckwheat bran powder with ultrafine grinding during *in vitro* gastrointestinal digestion. Capital letters indicate: the same group of Tartary buckwheat powder in different digestive conditions are compared, different letters differ significantly (*p* < 0.05). Lower case letters indicate: compare different groups of Tartary buckwheat flour under the same digestive conditions, different letters differ significantly (*p* < 0.05).

Figure 6 shows the change trend of antioxidative activity of TBP with different crushing frequency during *in vitro* intestinal digestion. After *in vitro* gastrointestinal digestion, the digestive juices of each group had a certain antioxidative activity, and the intestinal juice digestion group had the strongest antioxidative activity. The gastric juice digestion group had a significantly higher antioxidative activity than the gastric acid control group and the gastric blank control group. The antioxidative activity of TBP with different crushing frequency was slightly different during *in vitro* digestion. The scavenging capacity of hydroxyl-free radicals in gastric digestive fluid of the TBP, TBP-20, TBP-40, and TBP-60 was 58.52, 63.54, 69.17, 73.66%, and the scavenging capacity of hydroxyl radical in intestinal digestive fluid was 75.45, 79.95, 84.35 and 86.33%, respectively. The DPPH free radical scavenging ability of TBP with different crushing frequency was slightly different during *in vitro* digestion. The DPPH free radical scavenging ability in gastric digestive fluid of the TBP, TBP-20, TBP-40, and TBP-60 was 42.49, 46.67, 48.51, 50.31%, and the DPPH free radical scavenging ability in intestinal digestive fluid was 57.95, 62.86, 64.32, and 64.83%, respectively. Similarly, the ABTS free radical scavenging ability in gastric digestive fluid of the TBP, TBP-20, TBP-40, and TBP-60 was 60.56, 68.89, 71.64, 73.23%, and the ABTS free radical scavenging ability in intestinal digestive fluid was 80.53, 86.78, 89.64, and 91.64%, respectively.

After the TBP was ultrafine grinded, the dissolution rate of various active components increased, the antioxidant components in the solution increased, and the antioxidant capacity was improved (34). The simulated gastric juice was an acidic environment, and the hydrogen bond was unstable and easy to break under acidic conditions, which was conducived to the release of phenolic in complex (35). Previous study reported that during *in vitro* gastrointestinal digestion process of wheat bread, the amount of flavonoids released in intestinal digestion was higher than that in gastric digestion, and the antioxidant ability in intestinal digestive fluid was better than that in gastric digestive fluid, and the amount of flavonoids released was consistent with the trend of antioxidant capacity (36).

### 3.7. Correlation analysis

Table 1 shows the correlation between the release of flavonoids and the scavenging ability of hydroxyl free radical, DPPH free radical and ABTS cationic free radical during *in vitro* digestion of TBP with different crushing frequency. During *in vitro* gastrointestinal digestion, the amount of flavonoids released from digestive fluid was significantly correlated with antioxidant activity (*p* < 0.01), indicating that the more flavonoids released in digestive fluid, the stronger its antioxidant activity.

**Table 1:** Correlation between flavonoid release of Tartary buckwheat bran powder and antioxidant activity during *in vitro* digestion.

Antioxidant	Correlation			
	TBP	TBP-20	TBP-40	TBP-60
Hydroxyl radical scavenging ability	0.88^**^	0.93^**^	0.94^**^	0.93^**^
DPPH radical scavenging ability	0.95^**^	0.94^**^	0.96^**^	0.95^**^
ABTS^+^ radical scavenging ability	0.84^**^	0.92^**^	0.95^**^	0.95^**^

^**^ indicates a significant correlation at the 0.01 level.

The antioxidant capacity of food was affected by the content, composition and structure of phenolic acids and flavonoids in raw materials, and showed different antioxidant activities (37). The composition, structure and composition of these active substances in food will change under the influence of various enzymes and acid–base environment in the body, and their physiological and biochemical activities will also change (38). Therefore, ultrafine grinding can improve the antioxidant activity of TBP during *in vitro* digestion, which expands the application of TBP in functional foods.

## 4. Conclusion

This work evaluated that the effect of ultrafine grinding on the physicochemical and digestive properties of Tartary buckwheat powder (whole powder, core powder and bran powder). Through air ultrafine grinding, the particle size of various types of Tartary buckwheat powder is reduced, and the particle size of Tartary Buckwheat powder reaches its minimum value when the gas flow ultrafine grinding treatment is at 60 Hz. The ultrafine grinding process also led to an increase in the specific surface area of Tartary buckwheat powder particles, and as the frequency of ultrafine grinding increased, the specific surface area exhibited a more pronounced enhancement. The cation exchange capacity and glucose binding capacity of Tartary buckwheat flour were enhanced after superfine grinding, surpassing those of Tartary buckwheat core flour and whole flour. Furthermore, ultrafine grinding has the potential to enhance the release and antioxidant properties of Tarbuckwheat bran powder during *in vitro* digestion. In summary, the physical properties and flavonoid release performance of Tartary buckwheat powder can be enhanced through ultrafine grinding, with high frequency air micropulverization demonstrating superior effects under experimental conditions. These findings provide a theoretical foundation for the development and utilization of Tartary buckwheat-based food products.

## Data availability statement

The raw data supporting the conclusions of this article will be made available by the authors, without undue reservation.

## Author contributions

XW: Project administration, Funding acquisition, Formal analysis, Writing – original draft. XZ: Validation, Formal analysis, Writing – review and editing. DZ: Supervision, Writing – review and editing, Reviewed, Funding acquisition.
